# Integration Preferences of Wildtype AAV-2 for Consensus Rep-Binding Sites at Numerous Loci in the Human Genome

**DOI:** 10.1371/journal.ppat.1000985

**Published:** 2010-07-08

**Authors:** Daniela Hüser, Andreas Gogol-Döring, Timo Lutter, Stefan Weger, Kerstin Winter, Eva-Maria Hammer, Toni Cathomen, Knut Reinert, Regine Heilbronn

**Affiliations:** 1 Institute of Virology, Campus Benjamin Franklin, Charité-Universitätsmedizin Berlin, Berlin, Germany; 2 Institute for Computer Science, Freie Universität Berlin, Berlin, Germany; 3 Department of Experimental Hematology, Hannover Medical School, Hannover, Germany; Duke University Medical Center, United States of America

## Abstract

Adeno-associated virus type 2 (AAV) is known to establish latency by preferential integration in human chromosome 19q13.42. The AAV non-structural protein Rep appears to target a site called AAVS1 by simultaneously binding to Rep-binding sites (RBS) present on the AAV genome and within AAVS1. In the absence of Rep, as is the case with AAV vectors, chromosomal integration is rare and random. For a genome-wide survey of wildtype AAV integration a linker-selection-mediated (LSM)-PCR strategy was designed to retrieve AAV-chromosomal junctions. DNA sequence determination revealed wildtype AAV integration sites scattered over the entire human genome. The bioinformatic analysis of these integration sites compared to those of *rep*-deficient AAV vectors revealed a highly significant overrepresentation of integration events near to consensus RBS. Integration hotspots included AAVS1 with 10% of total events. Novel hotspots near consensus RBS were identified on chromosome 5p13.3 denoted AAVS2 and on chromsome 3p24.3 denoted AAVS3. AAVS2 displayed seven independent junctions clustered within only 14 bp of a consensus RBS which proved to bind Rep *in vitro* similar to the RBS in AAVS3. Expression of Rep in the presence of *rep*-deficient AAV vectors shifted targeting preferences from random integration back to the neighbourhood of consensus RBS at hotspots and numerous additional sites in the human genome. In summary, targeted AAV integration is not as specific for AAVS1 as previously assumed. Rather, Rep targets AAV to integrate into open chromatin regions in the reach of various, consensus RBS homologues in the human genome.

## Author Summary

This is the first unbiased genome-wide analysis of wildtype AAV integration combined with a thorough bioinformatic analysis of preferred genomic motifs and patterns in the neighbourhood of the integration sites identified. The preference of Rep-dependent AAV integration near multiple consensus Rep-binding sites was lost in the case of AAV vector integration in the absence of Rep expression. Our findings challenge the commonly accepted notion of site-specific AAV targeting to AAVS1 on chromosome 19q13.42. Although AAVS1 contains a canonical Rep-binding site, numerous additional sites including the newly identified hotspots AAVS2 on chromosome 5p13.3 and AAVS3 on chromosome 3p24.3 harbour functional Rep-binding sites suitable for AAV integration. AAV vectors are quickly moving forward in the clinic and Rep-dependent vector targeting strategies are being actively pursued. Detailed information of AAV wildtype versus recombinant AAV vector integration sites and preferences are needed to evaluate the safety profile of AAV vectors in gene therapy.

## Introduction

The family of adeno-associated virus (AAV) represents defective, helper-dependent viruses that need to establish latency to ensure persistence in their primate hosts [1]. Upon natural infections in humans AAV genomes were shown to persist mainly as episomes and integrated AAV genomes were rarely detected [2]. The molecular mechanisms leading to integration have only been characterized for AAV type 2 that prefers integration near a site on human chromosome 19q13.42, called AAVS1 [3]. The specificity of AAV integration is mediated by the large regulatory AAV proteins, Rep78/68 [4]. During productive AAV replication in the presence of either adeno- or herpesvirus as a helper virus, Rep78/68 is required for AAV gene expression and DNA replication. The AAV origins of DNA replication reside in the 145 bp inverted terminal repeats (ITRs) that flank the 4.7 kb single-stranded AAV genome. Rep78 and/or Rep68 are expressed from the AAV p5 promoter and were shown to bind to the Rep-binding site (RBS) within the AAV-ITRs [5]. Rep unwinds the DNA and introduces a single-strand nick at the adjacent terminal resolution site (*trs*) [6]. The AAV-ITRs also serve as *cis* elements for chromosomal integration [4]. A RBS homologue present in the AAV p5 promoter was shown to mediate AAV integration in the absence of the ITRs [7]. DNA sequences homologous to the RBS and a nearby *trs* element were also found in AAVS1 [8], [9] and, *in vitro*, ternary complex formation of Rep68 with the AAV-ITR and AAVS1 was shown [10]. A 33 bp sequence of AAVS1 spanning the RBS and the *trs* element was sufficient to mediate AAV integration *in vivo* [4], [11]. AAV integrated at variable distances from the RBS in AAVS1 and sequence rearrangements were frequently found at AAV-chromosome junctions [8], [9], [12], [13], [14], [15]. Quantitative real-time PCR analysis of AAVS1-specific AAV-2 integration within hours after AAV-2 infection and at increasing MOIs showed that 10 to 20% of infected cells displayed AAV integration within a 4 kb region of AAVS1 on chromosome 19q13.42 [16], [17]. In AAV-infected and subsequently selected cell clones up to 80% of AAVS1-specific integration had been described before [18].

Although AAV has not been associated with disease in humans, it is well established that AAV Rep78/68 induces DNA damage, cell cycle arrest [19] and apoptosis [20]. In addition, AAV Rep interferes with helper adenovirus- [21] herpes simplex virus replication [22]. AAV holds much promise as a vector for gene therapy. As a rule, recombinant AAV vectors persist as non-integrated, nuclear episomes. AAV vectors lack the integration promoting *rep* gene and therefore only occasionally integrate into the host cell genome. The preferred integration of wildtype AAV-2 in chromosome 19q13.42 is unique and is commonly viewed as a specifically evolved virus-encoded targeting mechanism. Multiple attempts were published that aim to exploit Rep-mediated targeting specificity for chromosome 19q13.42 for the specific integration of gene therapy vectors [23], [24], [25], [26], [27], [28]. Yet chromosome 19q13.42 is not the only target region. The presence of alternative integration sites has long been postulated and *in silico* analysis detected numerous consensus Rep-binding sites in the human genome. Many of these bound Rep *in vitro* [29] but their *in vivo* accessibility for AAV integration has not been explored so far. From an evolutionary standpoint the assumption that AAV latency is ensured by more than one target site or mechanism appeared reasonable.

This study was designed to close the knowledge gap between AAVS1-specific and assumedly non-AAVS1-specific wildtype AAV integration and to compare the identified genomic sites to those preferred upon AAV vector transduction. An open survey of chromosomal integration preferences for wildtype AAV-2 was conducted and complemented by the bioinformatic analysis of genomic motifs and patterns in the genomic regions surrounding the integration loci.

## Results

### General strategy of LSM-PCR

The genomic structure of latent AAV in infected cells is highly variable. Wildtype AAV-2 was shown to integrate into the host cell genome, as well as persist as extrachromosomal, nuclear episomes [2], [30]. In either case multicopy, concatemeric structures predominate and often lead to unpredictable rearrangements involving the 145 bp inverted terminal repeats (ITRs). Therefore the retrieval of AAV-chromosome junctions suffers from the inherent problem of inefficient PCR reads through the hairpin ITR into the adjacent chromosomal sequences. This leads to a predominance of rearranged AAV genomes lacking chromosomal junctions in previous PCR-based studies [31], [32], [33]. Furthermore, previously cloned junctions often displayed unknown intervening sequences of varying lengths between AAV and the identified chromosomal sequence [12], [15], [16], [27], [34], [35], [36]. Therefore, unambiguous assignment of the AAV-derived and chromosome-derived parts of junctions requires sufficient DNA sequence lengths.

**Figure 1:**
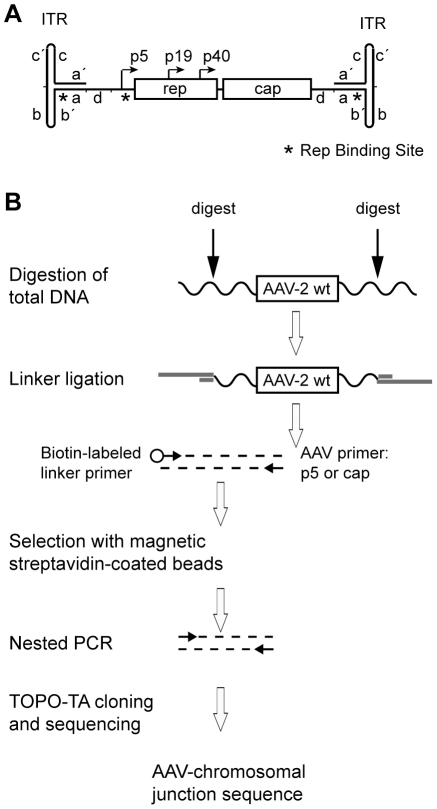
Linker-selection-mediated (LSM) PCR for cloning of chromosomal AAV integration sites. (A) Genome structure of AAV-2 with the *rep* and *cap* genes and their promoters flanked by inverted terminal repeats (ITRs) at either end of the ssDNA genome. The hairpin-structured ITRs contain internal repeat elements and complements thereof, represented by small letters. The positions of the Rep-binding sites (RBS) are represented by asterisks. (B) Schematic representation of the LSM-PCR strategy for amplification of AAV-chromosomal junction fragments. Wavy lines indicate chromosomal DNA. Linkers are displayed by thick, grey lines, AAV-specific primers by small, horizontal arrows. For restriction enzyme digestion indicated by vertical arrows either one of the following enzymes were used: PvuII or EcoRV (non-cut for AAV-2) or DraI (single-cut in AAV-2).

Several methods to identify virus-chromosome junctions have been developed to study retrovirus integration, where generally a single proviral copy per chromosomal site is found [37], [38]. The ultimate structure of the integrated long terminal repeat (LTR) is generally predictable in a way that allows an integration-specific PCR design. Linear amplification mediated (LAM)-PCR was initially designed to retrieve rare retroviral vector integration sites from small, clinical sample sizes [38]. We established a LAM-PCR with AAV primers in the “D” element of the AAV-ITR, the innermost and sole ITR region without internal inverse repetitions (Figure 1A). Unfortunately, pure AAV sequences with rearranged ITRs predominated, AAV-chromosome junctions were rare and the chromosomal DNA part often too short for unambiguous assignment to a unique genomic site. We then tested ligation-mediated (LM)-PCR that had been employed for broad surveys of lentivirus (HIV) or γ-retrovirus (MLV) integrations [39], [40], [41]. LM-PCR relies on a first LTR-specific primer. A linker is ligated to the first PCR strand that typically ends at the chosen restriction site within the unknown chromosomal sequence. A primer complementary to this linker ensures second strand synthesis and retrovirus-chromosome junctions are amplified by using a combination of retrovirus LTR-specific and linker-specific primer sets.

For this study a variation of LM-PCR, named linker-selection-mediated (LSM)-PCR was developed which enriched for *bona fide* AAV-chromosome fusion sequences. The genomic DNA of AAV-infected cells was cleaved with restriction enzymes that lead to sufficiently sized DNA segments to allow unambiguous genomic assignment of the chromosomal junction (Figure 1B). DNA sequences were amplified with one primer for a unique AAV-sequence, either of the p5 promoter or of the *cap* gene. The other primer binds to the linker DNA attached to the unknown chromosomal site. The structure of the linkers forces the PCR to initiate within the AAV genome, thereby suppressing amplification of chromosomal DNAs lacking integrated AAV. The use of non-cut enzymes for AAV-2 DNA helped to circumvent the problem of ligating linkers to episomal, non-integrated AAV DNA sequences. To further enrich for AAV-chromosome junctions a biotin tag was attached to the 5′-end of the linker primer. Thus, chromosome-derived PCR products could be enriched by streptavidin-mediated magnetic bead selection. This lead to PCR products selected for both, the presence of AAV and of an unknown chromosomal DNA sequence.

### AAV-2 integration sites

Using LSM-PCR a total of 1700 cloned PCR fragments were screened for DNA inserts of a minimal fragment size (>500 bp) to insure unambiguous detection of AAV-chromosome junctions. Out of 350 DNA sequence runs a total of 129 unique junction sites could be assigned to the human genome. Of these, 109 fulfilled the criteria outlined in the methods for unambiguous assignment of a single chromosomal site. Junctions were retrieved with non-cut enzymes for AAV-2, PvuII or EcoRV or with DraI, which cuts once in AAV-2 DNA outside of the region covered by the PCR. In addition, 43 wildtype AAV-2 infected Hela-derived single cell clones were generated of which eight harboured AAV-chromosome junctions that fulfilled the criteria outlined in the methods.

**Figure 2:**
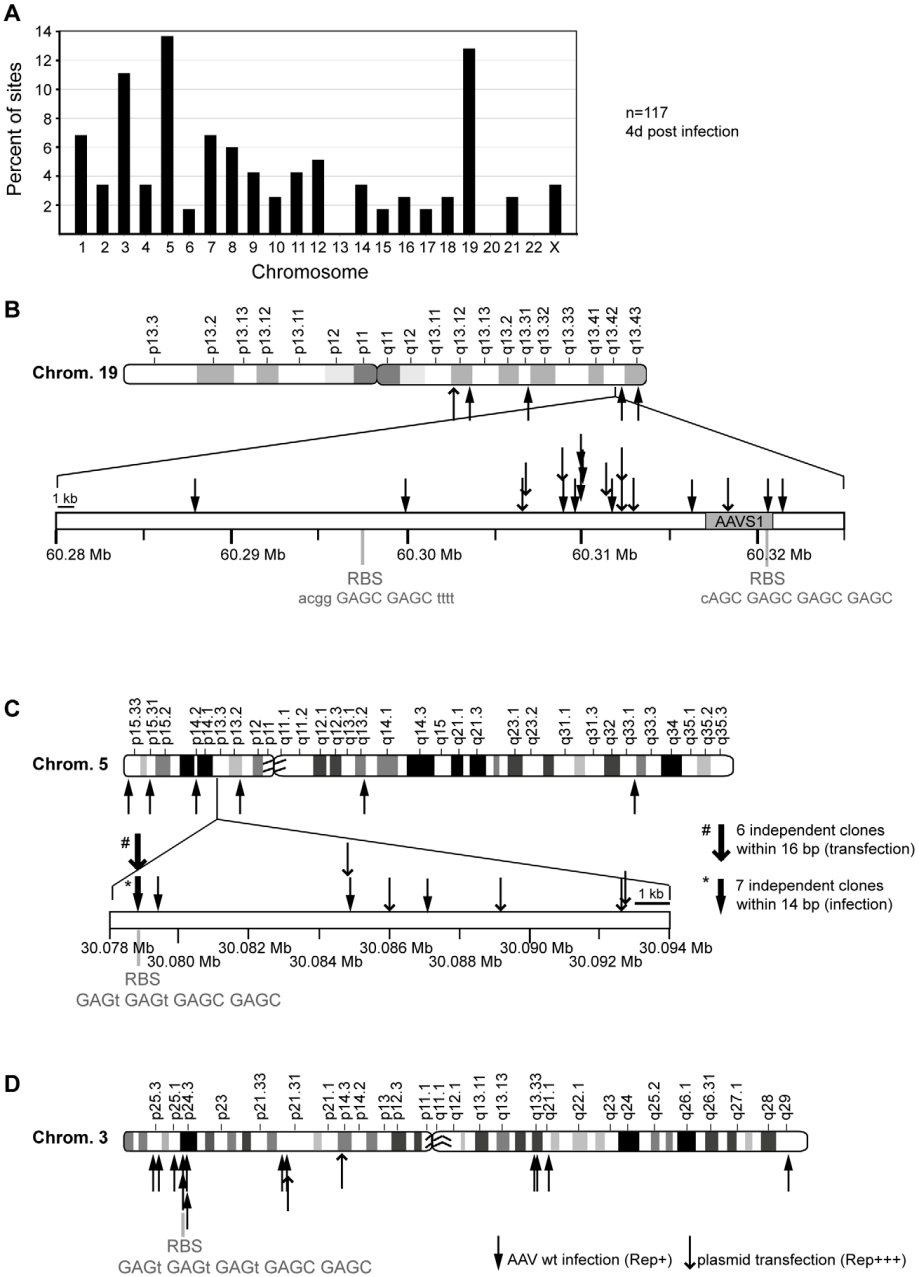
Chromosomal distribution of AAV-2 integration sites. DNA of AAV-2 wildtype infected Hela cells was analyzed for viral integration by the LSM-PCR-method. DNA sequence data from 117 cloned AAV-chromosome junctions were assigned to unique loci. (A) Distributions of junctions on individual chromosomes are shown as percentage of the total 117. (B–D) AAV integration sites drawn to scale for chromosome 19 (B), 5 (C), and 3 (D). Shown are the chromosome ideograms and enlarged bands of hotspots found on chr. 19q13.42 (AAVS1) and chr. 5p13.3 (AAVS2). Solid arrows represent sites detected upon wildtype AAV-2 infection. Open arrows represent junctions stemming from cotransfection of AAV vector- and Rep-expression plasmids.

DNA sequence analysis revealed that AAV-2 wildtype integration sites were scattered over the entire human genome. The chromosomal distribution pattern is displayed in Figure 2A. Over one third of AAV integration sites were clustered at hotspots on chr. 19q13.42, on chr. 5p13.3 and on chr. 3p24.3 (Figure 2B–D). Infection with AAV in the absence of a helper virus leads to transient, low Rep expression. Many previous AAV integration studies used plasmid transfections of wildtype or vector AAV constructs often in combination with a high-level Rep expression construct. To evaluate whether high Rep expression influenced the target site preference of AAV, the sequence data of previously published transfection-based AAV integration sites [42] were reevaluated with the more stringent criteria outlined in the method. Of 157 DNA sequences retrieved after cotransfection of a *rep*-expression construct and an AAV vector plasmid 47 junction sequences fulfilled our criteria for unambiguous assignment of AAV to a unique chromosomal site (Table 1).

**Table 1:** Summary of data sets analysed in this study.

Author	Reference	Source of integration sites	Number of junctions	Aim of study
Drew et al.	J Gen Virol, 2007	Cotransfection of *neo*-expressing AAV vector- and rep expression plasmids in HeLa cells. Selection of G418-resistent cell clones	47 (157)	Characterization of Rep78-dependent AAV-2 vector integration sites
Miller et al.	J Virol, 2005	AAV-2 vector infection of human diploid fibroblasts, no cell selection, analysis between 14–40 days p.i.	450 (1172)	Integration site pattern of AAV-2 vector integration (no rep)
Hüser et al.	This study	AAV-2 wt infection of HeLa cells, analysis at 4 days p.i.	109 a	Integration site pattern of wildtype AAV-2 integration
Hüser et al.	This study	AAV-2 wt infection of HeLa cells, expansion of single cell clones, no selection, analysis at 3–4 weeks p.i.	8 a	Integration site pattern of wildtype AAV-2 integration

Numbers in brackets represent the total numbers of junctions published in the given reference.

a Junctions derived from AAV-2 wildtype infected cells four days p.i. and from those after clonal expansion were combined for statistical analyses.

### Integration hotspots

For AAV wildtype 10% of all retrieved junctions were detected at the hotspot on chr. 19q13.42 spread over a total of 33 kb around AAVS1 (Figure 2B). Only one out of twelve chr. 19q13.42-specific AAV junctions was located within the 4 kb region of AAVS1, where a consensus Rep-binding site and an adjacent *trs* site had been defined [4] The reevaluated distribution pattern of junctions generated by transfection of AAV vector- and Rep expression plasmids [42] was similar (Figure 2B). Latently AAV-infected Detroit 6 cells [43], [44] were analyzed as control. Using cap-specific primers the junction was detected within AAVS1 at nucleotide position 60,319,992. A second hotspot named AAVS2 was detected on the small arm of chr. 5p13.3 within an intergenic region, where ten independent integration sites were detected within 8 kb (Figure 2C). In seven of these junctions clustered within 14 bp AAV had integrated directly into a consensus Rep binding site. The reanalyzed chromosomal integrations from AAV plasmid transfection [42] displayed a similar pattern with six integrations within 16 bp of the consensus RBS (Figure 2C). The third hotspot named AAVS3 was found on chr. 3p24.3 (Figure 2D). Out of 13 sites detected on chr. 3, three integrations were clustered in a 8 kb region where a consensus Rep binding site GAGT GAGT GAGT GAGC GAGC was detected on the complement strand (Figure 2D).

### Rep-binding affinity for RBS consensus sites in AAVS1,-S2, and S3

**Figure 3:**
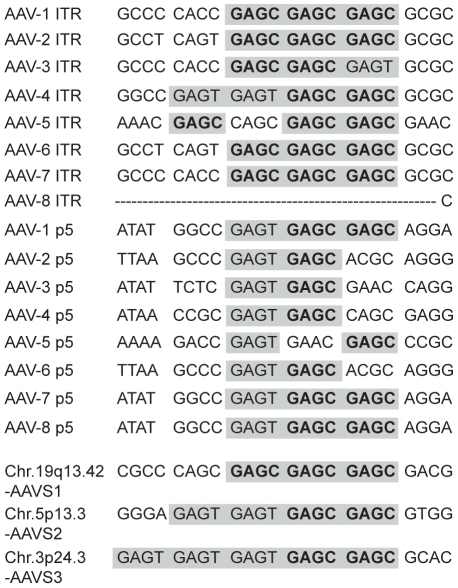
Sequence alignment of Rep-binding sites. RBS elements present in the ITR and the p5 promoter of all known AAV serotypes are aligned and related to consensus RBS sites present at chromosomal integration hotspots. Rep binding element GAGC is displayed in bold letters. Both GAGC and GAGT elements are highlighted in grey.

**Figure 4:**
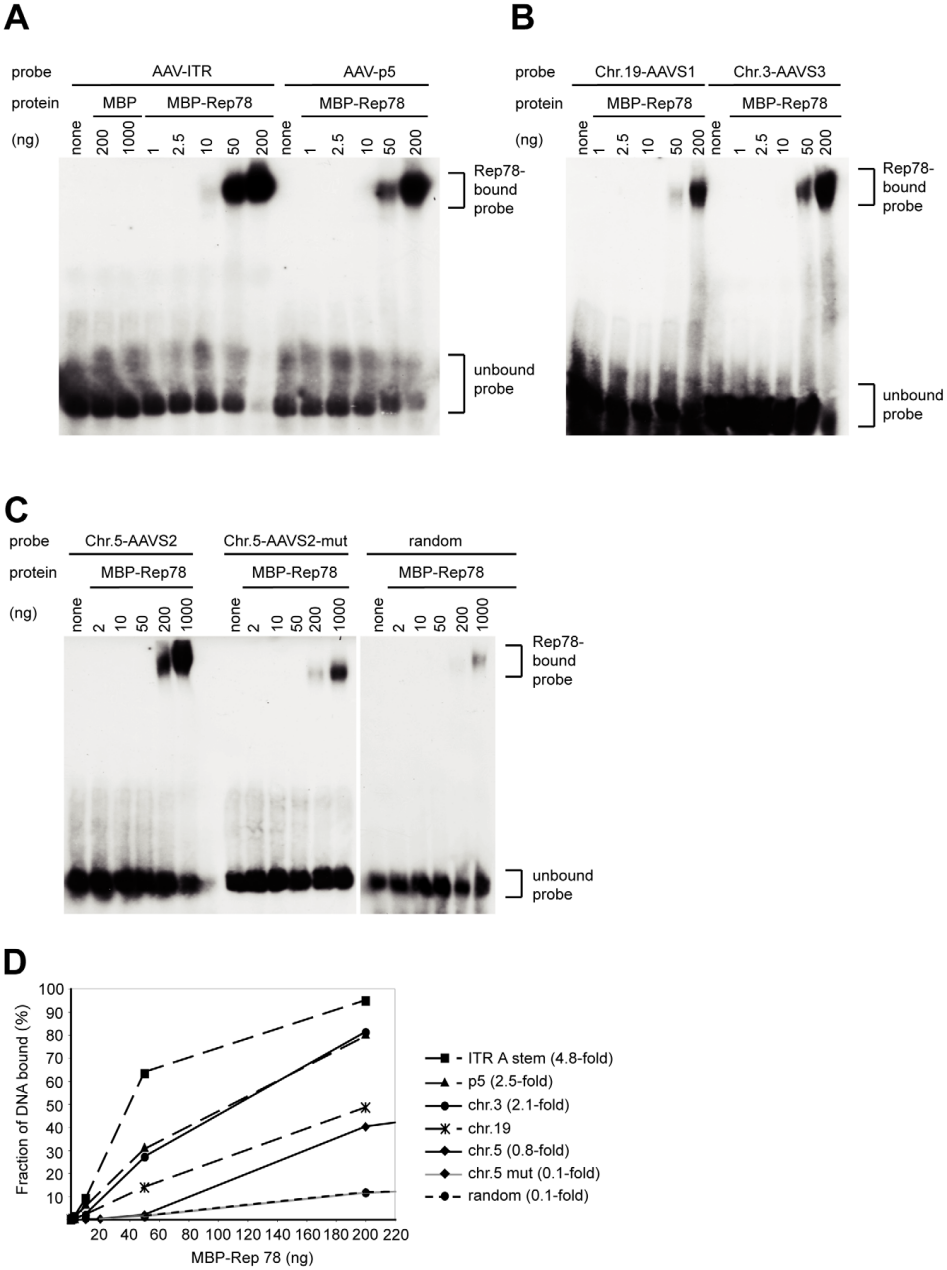
Binding of MBP-Rep78 to Rep-binding sites of AAV-2 and of chromosomal integration hotspots. (A) to (C) Electrophoretic mobility shift assays (EMSA) were performed with ^32^P-labeled double-stranded oligonucleotides in the presence of increasing amounts of affinity-purified MBP-Rep78 as indicated above the autoradiograms. (D) Quantitative determination of the bound fractions of the different RBS and control oligonucleotide probes as a function of the amount of MBP-Rep78 protein in the binding reaction. EMSA gels shown in (A) to (C) were subjected to phosphorimager analysis to determine the relative amount of unbound and bound ^32^P-labeled oligonucleotides. The relative binding affinity was calculated as follows: The highest amount of Rep used in this assay (1000 ng) bound 31% of the random oligonucleotide. The amount of Rep that bound the same fraction of the other oligonucleotides was determined and normalized to the binding of the chr. 19 (AAVS1) oligonucleotide.

To evaluate the binding affinities of Rep to the consensus RBS of the hotspots on chr.5 and chr. 3 compared to the RBS of chr. 19 or within the AAV genome, double-stranded oligonucleotides spanning the respective RBS regions (Figure 3) were submitted to mobility shift assays (EMSA) with increasing amounts of purified MBP-Rep78. Since it was previously shown that GAGG repeats are deficient in binding to Rep [10], [45], a mutated oligo derived from the RBS of AAVS2 displaying GAGG GAGG GAGC GAGG was used as a control. As an additional control, a random oligonucleotide of similar length was used. As shown in figure 4, the RBS of AAVS3 contained five instead of four GAGY repeats and bound Rep with a two-fold higher affinity than the oligonucleotide spanning the AAVS1 RBS and trs (Figure 4B). The RBS of AAVS2 showed 76% of the Rep-binding affinity of the AAVS1 sequence (Figure 4C). In contrast, the relative binding affinity normalized to the AAVS1 sequence dropped to 13% with the mutated AAVS2 oligonucleotide, which was in the range of the random oligonucleotide (Figure 4C). These findings confirm the importance of the GAGY repeats in Rep binding. As expected, Rep78 displayed the highest affinities for oligonucleotides spanning the A-stem of the AAV-ITR or the AAV p5–promoter (Figure 4A, 4D). In summary, the newly discovered hotspots for AAV integration, AAVS2 on chr. 5 and AAVS3 on chr. 3 display RBS similarly proficient for Rep-binding as AAVS1.

### Genomic features

To evaluate whether AAV-2 wildtype prefers specific motifs or genomic features for chromosomal integration the detected chromosomal junctions were compared to integration sites described for infection of human cells with a rep-deleted AAV-2 based vector [46]. The published DNA sequence files were reanalyzed using the criteria as outlined in the methods. This led to 450 junctions that could be included as an AAV vector-specific data set (Table 1). The preference for integration next to selected genomic features was analyzed for rep-positive AAV wildtype and for rep-deficient AAV vectors (Table 2). The data showed that the integration frequency of AAV wildtype in genes was higher than expected by chance (Table 2). The frequency was comparable to that of rep-deficient AAV vectors, thus confirming the findings by Miller et al. [46].

**Table 2:** Genomic features and chromatin state associated with AAV-2 wildtype- and AAV vector-derived integration sites.

Genomic feature	AAV wt infection % of sites (n = 117)	AAV vector infection % of sites (n = 450)	Random control % of sites	p-Value wt^a^	p-Value vector a
RefSec genes	50.4	51.8	40.1	<0.05	<0.01
RefSec genes tss+/−2 kb	6.8	5.6	2.9	<0.05	<0.01
Ens genes	51.1	51.3	40.7	<0.05	<0.0001
GenScan genes	83.8	74.0	69.5	<0.001	<0.05
Known genes	57.3	55.8	45.2	<0.01	<0.0001
Known genes exons	3.4	3.6	2.7	n.s.	n.s.
CpG	0.9	1.6	0.7	n.s.	<0.05
CpG+/−2 kb	7.7	6.4	4.1	n.s.	<0.05
Histone modification					
H3K4me1	13.7	n.d.	7.5	<0.05	n.d.
H3K4me3	6.8	n.d.	2.3	<0.01	n.d.
H3K27me3	28.2	n.d.	42.2	<0.01	n.d.

a P-values were determined in comparison to random controls, n.d.  =  not determined, p>0.05 were not considered statistically significant (n.s.).

### Chromatin state at AAV integration sites

To analyze the effect of epigenetic modifications on AAV integration the association of integration sites with histone modifications as markers for open or closed chromatin were assessed by chromatin immunoprecipitation sequencing (ChIP-Seq) analysis as outlined in the methods. Trimethylated lysine 27 of histone 3 (H3K27me3) is correlated with gene repression (closed chromatin) [47], while methylation of lysine 4 in H3K4me3 and H3K4me1 is indicative of promoter or enhancer regions (open chromatin) [48]. As shown in table 2 the association of AAV wildtype with open chromatin regions is significantly higher than expected from random controls. Conversely, the respective association with closed chromatin is significantly reduced. In summary, AAV wildtype prefers integration into open chromatin whereas closed chromatin was avoided.

### Bioinformatic analysis of the AAV integration sites

A series of publications have shown that fused combinations of two to four GAGC motifs bind to Rep78/68 of AAV-2 [4], [49], [50], [51], [52], [53]. Moreover, *in vitro* ternary complex formation of Rep68 with the AAV-2 ITR and AAVS1 of chr. 19q13.42 [10] led to the concept of Rep acting as an adapter that targets AAV to the human genome. Although only AAV-2 has been analyzed for chromosomal integration so far, all known AAV serotypes displayed various combinations of GAGC and/or GAGT motifs in the ITR and the p5 promoter. An alignment of these AAV elements to the integration hotspots AAVS1, AAVS2 and AAVS3 is displayed in Figure 3.

**Figure 5:**
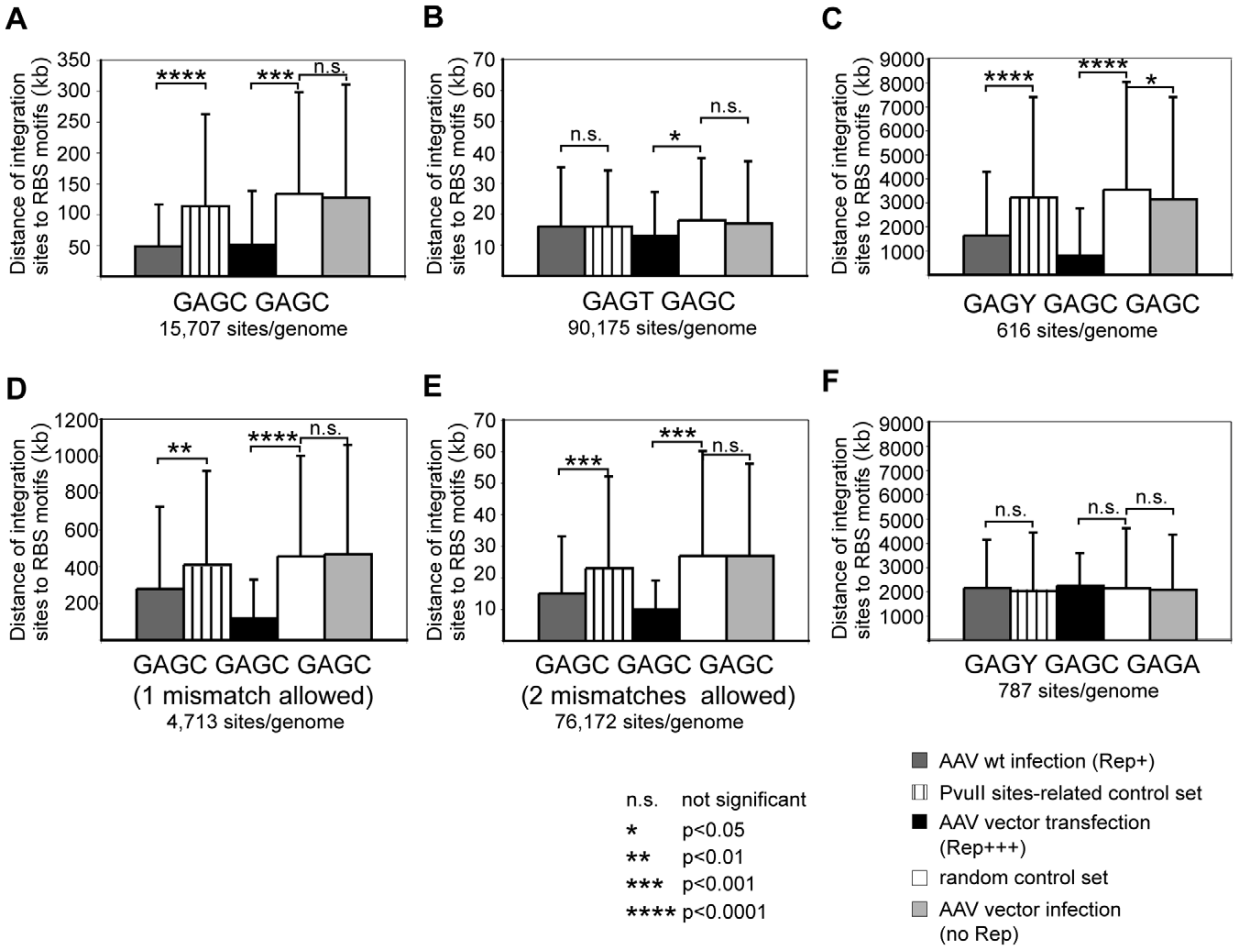
Statistical analysis of distances of integration sites from human Rep-binding motifs. AAV integration sites of different data sets were analyzed for the proximity to the next Rep binding sites. Calculations were performed with the following putative Rep binding sites: GAGC GAGC (A); GAGT GAGC (B); GAGY GAGC GAGC (C); GAGC GAGC GAGC, one mismatch (D), or two mismatches allowed (E), and GAGY GAGC GAGA (F). Average distances of integration sites from putative Rep binding sites are displayed as mean +/− S.D. The levels of significance are marked by asterisks. P-values >0.05 were not considered statistically significant. P-values <0.01 were considered highly significant. For the analysis of wildtype AAV integration data a PvuII-related control set was used in addition to the random control set.

Based on these data we hypothesized that AAV-2 wildtype, due to the presence of Rep, prefers integration at chromosomal sites in closer proximity to consensus Rep binding sites than would be expected from control sites. The hypothesis was tested with the three sets of junctions derived from: 1. Infection with AAV-2 wildtype, 2. Cotransfection of plasmids coding for an AAV vector and a constitutive Rep-expression cassette, and 3. Infection with Rep-deficient AAV vectors (Table 1). The distances between any one integration site and its nearest Rep-binding site were determined in the human genome and compared to similarly determined distances of individual control sites to the nearest Rep-binding sites. Calculations were repeated using various combinations of RBS as displayed in Figure 5.

The choice of randomly generated genomic control sites was considered optimal for comparative analysis of the three sets of data. Yet, a concern was the choice of restriction endonucleases for the identification of the wildtype AAV-2 integration sites by LSM-PCR. To control a bias introduced by a conceivable non-random genomic distribution of the restriction sites, the average distance of PvuII, EcoRV, or DraI-generated restriction sites to putative Rep-binding sites was compared to the average distances of random sites to Rep-binding sites. PvuII restriction sites were found to be closer to Rep-binding sites than random control sites (Figure S1). This was assumedly due to the high G+C content of the PvuII recognition sequence and of the consensus Rep-binding sites. Both EcoRV and DraI sites were found further apart from Rep-binding sites in accordance with their high A+T content (Figure S1). To circumvent any bias arising from the use of PvuII, the data set for AAV wildtype infection was calculated against the data set of random control sites as well as against the data sets for the restriction site–related controls. Since not more than two thirds of sites were generated with PvuII, the PvuII-related control sites would at most underestimate the association to Rep-binding sites and was therefore used as the most stringent control set. In addition all calculations were also performed with the set of random controls leading to similar findings (Figure S2).

The bioinformatic calculations with GAGC GAGC as a minimal Rep-binding site strikingly confirmed our hypothesis that integration of wildtype AAV takes place close to Rep-binding sites with very high significance (p <0.0001). A comparable effect was seen with the data set for AAV vectors in the presence of Rep (p<0.001). Most importantly, the set of integration sites for AAV vectors in the absence of Rep did not show any difference of integration site preference compared to random control sites (Figure 5A). With a frequency of 15,707 sites per human genome the Rep binding motif GAGC GAGC occurs sufficiently frequent to lead to a mean distance of around 50 kb to the next AAV integration site in the presence of Rep. In the absence of Rep the mean distance to AAV (vector) integration sites rises to around 130 kb (Figure 5A). To ensure that the presence of repetitive DNA in the random controls did not lead to a bias in the analysis, an independent control calculation was performed for AAV wt data using AAV vector infection data as background. The high significance level was maintained (data not shown). The significance of the Rep-associated preferential integration near GAGC GAGC sequences was further underlined by the results of similar calculations for the putative Rep-binding motif GAGT GAGC, where no such association was found. Only in the presence of presumably large amounts of Rep (AAV vector transfection, Rep+++) a small effect was seen (Figure 5B). Obviously the GAGT GAGC motif is not sufficient to attract Rep and the AAV genome for integration. When an additional GAGC repeat is added (GAGY GAGC GAGC) the integration preferences of AAV wildtype and Rep-expressing AAV vectors shifted to closer proximity to Rep-binding sites (p<0.0001). This is especially surprising since only 616 sites per human genome are found for GAGY GAGC GAGC (Figure 5C). To allow more potential Rep-binding site permutations, calculations were repeated with the consensus GAGC GAGC GAGC with one or two random mismatches. This led to a significantly decreased mean distance to AAV junctions in spite of the fact that up to 100-fold more genomic hits were found for the motifs (Figure 5D; E). A single nucleotide exchange in the GAGY GAGC GAGC motif (Figure 5F, GAGY GAGC GAGA ) on the other hand led to a complete loss of association to AAV integration sites. This is surprising in view of the reported *in vitro* binding of Rep to this motif [45] and supports the assumption that the C at the 3′ end of the Rep binding motif is relevant for Rep-binding *in vivo*. Motifs GCCC GAGT GAGC and GAGT GAGC ACGC are part of the RBS in the viral p5 promoter. The individual motifs are found at very low frequency (n = 85, or n = 82, respectively) in the human genome, so that either no RBS was found in the same contig or the distance to the next RBS was more than several thousands kb. For these reasons we did not proceed with calculations for these motifs. To further exclude the possibility that the calculated associations with Rep binding sites were predominantly based on sequences assigned to the hotspots AAVS1 and AAVS2, the significance of the associations was re-evaluated with data sets omitted for the hotspot sequences (Table 3). The robustness of the data becomes evident by the fact that the highly significant association of AAV junctions to motifs GAGC GAGC and GAGY GAGC GAGC is maintained. In summary, AAV prefers integration sites in the vicinity of consensus Rep-binding elements, most prominently on chr. 19q13.42 (AAVS1), chr. 5p13.3 (AAVS2), and chr. 3p24.3 (AAVS3). But even in the absence of hotspots AAV still shows a highly significant integration preference for Rep-binding motifs at numerous additional sites in the human genome.

**Table 3:** Neighbourhood analysis of wildtype AAV-2 integration sites and RBS motifs found outside of the hotspots on chr. 19q13.42 (AAVS1) and on chr. 5p13.3 (AAVS2).

RBS motif	all integration sites (n = 117)	no chr.19q13.42 (n = 106)	no chr.19q13.42 no chr.5p13.3 (n = 96)
GAGC GAGC	<0.0001	<0.0001	<0.001
GAGY GAGC GAGC	<0.0001	<0.001	<0.01
GAGC GAGC GAGC (1 mismatch allowed)	<0.01	<0.05	not significant a
GAGC GAGC GAGC (2 mismatches allowed)	<0.001	<0.01	<0.05

Displayed are p-values calculated with the PvuII control set.

a p-values >0.05 were not considered statistically significant.

## Discussion

This study represents the first genome-wide survey of wildtype AAV-2 integration in the human genome combined with a thorough bioinformatic analysis of the surrounding genome. We show here that wildtype AAV-2 infection leads to preferential integration in the vicinity of consensus Rep-binding sites (RBS) at defined hotspots as well as at numerous additional genomic sites. In contrast, AAV-2 vectors in the absence of Rep-expression integrate without discernable preference for consensus Rep-binding sites.

### Hotspots of AAV integration

At the hotspot on chr. 19q13.42, up to 10% of all AAV junctions were scattered over a region of 33 kb, mostly in centromeric direction with regard to the previously defined core 4 kb AAVS1 site. AAV vectors in the absence of Rep expression do not show any preference for chr. 19q13.42 [46]. The here identified, novel hotspot AAVS2 on chr. 5p13.3 displayed roughly 8% of all junctions retrieved from wildtype AAV-2 infection and 23% of those retrieved from cotransfection of AAV vectors in the presence of Rep distributed over a region of 14 kb. A cluster of 13 independent junctions was found within 14 bp of the AAVS2 RBS that was shown to be similarly proficient in binding to Rep *in vitro* as is the RBS of AAVS1 (Figure 4). The high *in vivo* integration numbers may in part be due to the choice of HeLa as target cells. These are hypertriploid with up to 12 copies of the p-arm of chr. 5 [54]. The extra gain of integrations within the described 8 kb region is however unique for the AAVS2 site and not accompanied by a parallel increase of integrations at additional sites on the overrepresented p-arm of chr. 5, where 201 additional GAGC GAGC repeats and three additional GAGY GAGC GAGC repeats were counted. The only fourfold tetranucleotide repeat on the chr.5 p-arm is found in AAVS2 (GAGT GAGT GAGC GAGC; Figure 2C). In addition, junctions of rep-deficient AAV vector were reported to be underrepresented on chr. 5 [46].

A major difference between the hotspots on chr. 5 and chr. 19 concerns the presence of genes. The junctions identified on chr. 19 span the region of the transcribed gene for protein phosphatase 1, regulatory subunit 12C (*PPP1R12C*). The 8 kb AAVS2 sequence identified on chr. 5p13.3 represents an intergenic region to the best of current knowledge. It is well known that Rep expression leads to extensive rearrangements of AAVS1 [18], [55], [56]. Apparently, *PPP1R12C* is essential, since the majority of latently infected cell lines display gene duplications [57] and simultaneous AAV integrations in both alleles have never been reported. A currently unresolved question concerns the presence of a terminal resolution site (*trs*) next to the RBS of AAVS2 and AAVS3. In AAVS1 the spatial configuration of RBS and *trs* resembles that of the AAV-ITR. The *trs* element lies next to the RBS and serves as a nicking site for Rep [4]. In AAVS2 and AAVS3 the nearest perfect *trs* elements (5′-GTTGG-3′) are 400 and 500 bp away from the RBS, which represents the mean statistical occurrence for this motif. Unfortunately, the consensus nucleotide requirements for a functional *trs* element are not defined well enough to conduct a meaningful bioinformatic search. Therefore, the presence of nicking sites next to the RBS in AAVS2 or AAVS3 remains open at present.

### Target site choice for AAV integration

Besides the identified integration hotspots numerous additional chromosomal junction sites were found for integrated wildtype AAV-2, scattered over the human genome. From the bioinformatic calculations it appeared that the perfect tetranucleotide repeat GAGC GAGC represented the minimal requirement for Rep-dependent targeted integration, and GAGY GAGC GAGC represents the optimized *in vivo* target sequence for wildtype AAV-2. Hotspots AAVS1, AAVS2, and AAVS3 display this core sequence fused to additional imperfect GAGY repeats. Other AAV serotypes display RBS sequences with similar numbers of GAGC and/or GAGT repeats, extended by additional imperfect repeats. AAV5 Rep co-crystallised with the hairpin-structured AAV5-ITR revealed that five Rep monomers bind to five consensus tetranucleotide repeats of the RBS, each of which was contacted by two Rep monomers from opposite faces of the DNA [58]. AAV2-Rep78/68 was shown to simultaneously bind to the RBS of the AAV-2 ITR and to that of AAVS1 [10]. Although it is currently unknown whether other AAV serotypes integrate at all, this is highly likely in view of the ability of both AAV-2 Rep and the relatively distant AAV-5 Rep to multimerize and simultaneously bind to clustered GAGY repeats.

In the initial descriptions of AAVS1, site-specific nicking of the *trs* by Rep bound to the adjacent RBS was viewed as preferred entry site for AAV recombination [4]. Meanwhile the majority of AAV integrations on chr. 19q13.42 were found many kb away from the RBS-*trs* combination, and neither AAVS2 or AAVS3 display obvious *trs* homologues next to the RBS. Therefore alternative explanations for RBS-dependent AAV integration should be considered. The potential use of preexisting chromosomal breakage sites recalls a mechanism already proposed for the integration of rep-deficient AAV vectors [34], [59]. Alternative integration concepts include the use of imperfect *trs* elements for nicking as shown *in vitro* [4], [60], [61], or the ability of Rep78 to induce DNA damage *in vivo* by single-strand nicking of cellular chromatin [19]. It is conceivable that the introduction of single-strand nicks occurs anywhere in accessible chromatin, even if the nicking site is hundreds or thousands of bp apart from the RBS on an extended DNA strand. HMGB1, an ubiquitous architectural protein that serves as key component of the chromatin remodelling complex may be of help [62]. Its long-known *in vivo* interaction with Rep [63] may help remodel the chromatin to make it accessible for nicking by Rep. Rep was also shown to contact other key players of the nucleosome remodelling complex as components of the transcription- or DNA replication machinery [64], [65], [66]. Any of these mechanisms can be exploited to open the chromatin for AAV integration. In summary, Rep with its combined DNA-binding and endonuclease activity appears to serve as a relatively imprecise targeting tool for AAV integration preferably in open chromatin regions in the reach of consensus Rep-binding sites prevalent in the human genome.

### Implications for Rep-dependent targeting of AAV vector integration

The early finding that Rep would mediate site-specific AAV integration on chr. 19q13.42 had immediate implications for gene therapy. A variety of concepts were devised to incorporate Rep as an adapter to target AAV-ITR flanked transgenes to a specific site [26], [27], [28], [57], [67]. In the majority of cases appropriate cell selection or PCR for AAVS1 led to cells displaying the desired integration. The reported high frequencies of integration into AAVS1 are difficult to reconcile with our findings, unless the level of Rep expression is considered to have an impact on target site choice. Upon AAV infection Rep is only moderately expressed due to autoregulation of the AAV p5 promoter. Rep-dependent AAV vector transductions typically use strong heterologous promoters that lead to high and sustained Rep expression levels. Increasing Rep levels may increase the overall probability for integration anywhere in the genome, including at hotspots. Under these conditions AAVS1-specific integration will be detected more readily. This appears however to come at the price of genomic rearrangements in reach of alternative Rep-binding sites. Therefore, it is plausible that in the absence of any selection AAV integration into AAVS1 is typically unstable and difficult to detect.

In summary, Rep expression increases the probability for integration next to one of several genomic hotspots. However, the net genotoxic effect is unpredictable both with respect to the integrity of the AAV integration locus itself and with respect to the numerous additional sites where Rep binds and initiates chromosomal damage. Therefore, the current concept of a relatively precise site-specific targeting of AAV should be extended to a concept of a relative preference for accessible chromatin regions in the neighbourhood of any of the numerous consensus Rep-binding sites. More recent approaches for site-specific vector targeting try to exploit DNA sequence-specific zinc-finger nucleases to target a genomic sequence of wish [68]. Although zinc-finger nucleases are not free from off-target genotoxicity, at least the genomic targeting site for the transgene can be more precisely defined, a goal that appears to be inherently unachievable using Rep as an adapter molecule.

## Materials and Methods

### Cells

Detroit 6 cells harbouring latent AAV-2 genomes and HeLa cells were grown in Dulbecco's modified Eagles's medium (Gibco) supplemented with 10% fetal calf serum, penicillin (100 U/ml), and streptomycin (100 µg/ml).

### AAV infection

Viral stocks of wildtype AAV-2 with infectious titers of 5×10^9^ i.u./ml were prepared on HeLa cells as described before [16]. For the analysis of AAV integration sites 1.7×10^6^ HeLa cells were seeded overnight on 10 cm diameter dishes and infected with AAV-2 at a MOI of 500. Cells were harvested at 96 hours post infection (p.i.) for the extraction of genomic DNA. The period of cell growth after infection was minimized to reduce the chances of selection of particular integration sites during cell proliferation. Alternatively, AAV-infected HeLa cells were seeded to microtiter plates at a dilution of 60 cells per plate and grown up as single-cell clones without drug selection.

### Plasmids

Plasmid pTAV2-0 covers the AAV-2 wildtype genome (GenBank accession number AF043303), pRVK the 4 kb fragment of the AAVS1 locus on chromosome 19 (GenBank accession number S51329), and pAAVS1-TR covers an AAV-ITR/AAVS1 junction [16]. Plasmid pMBP-Rep78 encoding Rep78 fused to maltose-binding protein (MBP) was described before [69].

### Production and purification of MBP-Rep78 fusion protein

MBP-Rep78 encoding Rep78 fused to maltose-binding protein was expressed und purified essentially as described [69]. Briefly, E.coli strain BL21 transformed with pMBP-Rep78 was grown at 30°C to an OD_600 nm_ of 0.6 to 0.8. Production of MBP-Rep78 was induced with 0.3 mM IPTG for 3 h at 30°C. Cells were harvested by centrifugation and lysed by sonication for 2 min (30% duty cycle) in lysis buffer of 50 mM phosphate pH 7.8, 300 mM NaCl, 1% (v/v) Triton X-100, 0.1 mM PMSF. Cell debris was removed by centrifugation at 6500×g for 20 min at 4°C. The supernatant was adsorbed to amylose resin (New England Biolabs) in a batch process and the resin was washed extensively (5 washes with about 100 volumes of the resin) with lysis buffer. The adsorbed proteins were eluted with lysis buffer containing 10 mM maltose and analyzed for purity by SDS-polyacrylamide gel electrophoresis.

### Electophoretic mobility shift assays (EMSA)

Binding of MPB-Rep78 fusion protein to ^32^P- labeled double-stranded oligonucleotide probes was detected by altered mobility of the probes in nondenaturating polyacrylamide gels essentially as described previously [70]. Briefly, oligonucleotides of 46–49 nt length were end-labeled with T4 polynucleotide kinase and annealed. EMSA reactions were performed for 20 min at 20°C as follows: 0.015 pmol of labeled DNA substrate was incubated with the indicated amounts of MBP or MBP-Rep78 in a binding buffer containing 25 mM HEPES-KOH (pH 7.8), 10 mM MgCl_2_, 40 mM NaCl, 1 mM DTT, 2% glycerol, 12.5 µg/ml BSA, 0,01% Nonidet P40 and 5 µg/ml salmon sperm DNA. The following oligonucleotides were used:

AAV-ITR (nucleotide position 85–133): GCCTCAGTGAGCGAGCGAGCGCGCAGAGAGGGAGTGGCCAACTCCATCA;

AAV-ITR complementary strand: TGATGGAGTTGGCCACTCCCTCTCTGCGCGCTCGCTCGCTCACTGAGGC

Chr. 19q13.42 (AAVS1): TGGCGGCGGTTGGGGCTCGGCGCTCGCTCGCTCGCTGGGCGGGCGGGC

Chr19 (AAVS1) complementary strand: GCCCGCCCGCCCAGCGAGCGAGCGAGCGCCGAGCCCCAACCGCCGCCA

Chr. 5p13.3 (AAVS2): AGCTGGACCCCACGCTCGCTCACTCACTCTCCCCTCACCGCTTTGT

Chr. 5 (AAVS2) complementary strand: ACAAAGCGGTGAGGGGAGAGTGAGTGAGCGAGCGTGGGGTCCAGCT

Chr. 3p24.3 (AAVS3) GCTTCCCAAGGGGAATGAATGTGCGCTCGCTCACTCACTCACTCCTCAC

Chr.3 (AAVS3) complementary strand: GTGAGGAGTGAGTGAGTGAGCGAGCGCACATTCATTCCCCTTGGGAAGC

Chr. 5MUT (AAVS2 mutated): AGCTGGACCCCAC**C**CTCGCTC**C**CTC**C**CTCTCCCCTCACCGCTTTGT

Chr.5MUT (AAVS2 mutated), complementary strand: ACAAAGCGGTGAGGGGAGAG**G**GAG**G**GAGCGAG**G**GTGGGGTCCAGCT

AAV p5 (nucleotide position 245–292): TCACGCTGGGTATTTAAGCCCGAGTGAGCACGCAGGGTCTCCATTTTG

AAV p5 complementary strand: CAAAATGGAGACCCTGCGTGCTCACTCGGGCTTAAATACCCAGCGTGA

random control: CAGAGCAGCAGCACAGACGCTAGCAGATCTCCTGCGACCGGAGATGTG

random control, complementary strand: CACATCTCCGGTCGCAGGAGATCTGCTAGCGTCTGTGCTGCTGCTCTG

### Preparation of genomic DNA

Total genomic DNA was extracted by SDS/proteinase K digestion followed by repeated phenol/chloroform extractions and ethanol precipitation, as described before [71]. High molecular weight DNA (2 µg) was digested with restriction enzymes that lead to a mean genomic fragment size of around 4 kb and produce blunt-ends ready for linker/adapter ligation. Non-cut enzymes for AAV-2 DNA were preferred, PvuII, EcoRV. Additional junctions were retrieved with DraI (one cut in AAV-2 wildtype DNA). Digested genomic DNA was purified by repeated phenol-chloroform extractions and precipitated with ethanol.

### Linker-Selection-Mediated (LSM) PCR

A linker-based strategy described in [39], [40] and outlined in more detail in the manual of the GenomeWalker kit (Clontech) was modified as outlined in Figure 1B. The following oligos were used for linker construction: “Linkerlong” (5′GTA ATA CGA CTC ACT ATA CGG CAC GCG TGG TCG ACG GCC CGG GCT GGT 3′) and “linkershort” (5′ACC AGC CC 3′modifikation: 2′,3′-dideoxyC). Equal amounts of “linkerlong” and phosphorylated “linkershort” (100pmol each) were annealed and ligated to restriction enzyme-digested genomic DNA.

PCR-primers: The linker-primers were “P linker outside” with biotin attached to its 5′ end (5′-GTA ATA CGA CTC ACT ATA CGG C; T_m_ = 58.4°C) and “P linker nested” (5′-ACT ATA CGG CAC GCG TGG T; T_m_ = 58.8°C). Two AAV-2-specific primer sets were used. The first primer set covered the AAV p5 promoter: “AAV2p5” (5′-TCA AAA TGG AGA CCC TGC GTG CTC A; T_m_ = 64.6°C, AAV-2, nt 293–269), primer “AAV2p5 nested” (5′-TAA ATA CCC AGC GTG ACC ACA TGG TG; T_m_ = 64.8°C, AAV-2, nt 260–235). The other primer set is located in the *cap* gene region, as described before [2]: “CAPgsp1” (5′-GTC TGT TAA TGT GGA CTT TAC TGT GGA CAC; T_m_ = 65.4°C, AAV-2, nt 4320–4349) and “CAPgsp2” 5′-GTG TAT TCA GAG CCT CGC CCC AT; T_m_ = 64.2°C, AAV-2 nt 4357–4379).

The PCR reaction contained 0.2 mM dNTPs, linker primer and AAV specific primer (0.25 µM, each), 2.5 U proofreading hot-start polymerase (Herculase) in reaction buffer, as provided by the supplier (Stratagene). Of the preceding linker-ligation reaction 1–5 µl was added to a final volume of 50 µl. PCR conditions were as follows: 3 min at 98°C, followed by 10 cycles of 40 sec at 98°C, 30 sec at 65°C, and 4 min at 72°C, followed by 20 cycles of 40 sec at 98°C, 30 sec at 65°C, and 4 min + 10 sec per cycle at 72°C, terminated by an extension period of 10 min at 72°C. Biotin-labelled PCR products were further enriched on streptavidin-labelled Dynabeads M-280, as outlined by the supplier (Invitrogen). Subsequent nested PCR used conditions identical to the first round but with pairs of the nested PCR primers, as outlined above. Finally, to add overhangs of multiple As, PCR products were incubated with 1 U Taq polymerase (New England Biolabs).

### Analysis of LSM PCR products

Products of LSM-PCR reactions were separated on agarose gels. To ensure sufficient chromosomal fragment lengths, PCR bands of a calculated minimal length (>500 bp) were excised and purified by the QIAEX II Gel extraction kit (Qiagen, Hilden, Germany). TOPO-TA cloning was performed as described [72]. Colonies were PCR-screened with the M13 forward (-20) and reverse primer pair (0.4 µM, each) with 0.2 mM dNTP, 2 U Taq polymerase (New England Biolabs) at the following conditions: 10 min at 94°C, followed by 30 cycles of 30 sec each at 94°C, 52°C, and 72°C, followed by 10 min at 72°C. Column-purified PCR products were submitted to DNA sequencing using the primer provided by the TOPO-TA cloning kit. DNA sequences were run on a CEQ2000 genetic analysis system (Beckman) using the CEQ Dye Terminator Cycle Sequencing Quick start kit (Beckman) and the run method LFR-a. Cycling conditions were as follows: 1 min at 96°C, followed by 30 cycles 20 sec at 96°C, 20 sec at 50°C and 4 min at 60°C.

### Integration site determination

The genomic positions of AAV integration sites in the human genome (assembly from March 2006, hg18) were determined using the BLAT tool from the UCSC Genome Browser web site (http://genome.ucsc.edu/cgi-bin/hgBlat) [73]. A match was defined as a BLAT search result fulfilling all of the following criteria:

1. A human chromosome-derived part of the DNA sequence is at least 100 bp in length and of 98% or higher homology to the database.
2. A shorter chromosomal match is acceptable if it displays a minimum of 25 bp of a contiguous DNA sequence match.
3. A part of the sequence allows assignment of AAV.
4. In the case of unassigned base pairs between the AAV and the human part of the sequence, this sequence is no longer than 20 bp.
5. Sequences matching to multiple chromosomal regions (i.e. repeat regions) were discarded in view of the inability to unambiguously assign the surrounding genome for subsequent bioinformatic analysis (see below).
6. Duplicate AAV-chromosomal fusion sequences (identical viral and identical human DNA sequences) were counted only once.

In addition to the LSM-PCR derived sequences, the original DNA sequence files of 157 chromosomal junctions [42] kindly provided by Dr. G.W. Both, North Ride, Australia were reanalyzed applying the above inclusion criteria. This led to 47 DNA sequences suitable for our analysis (Table 1). In their study, HeLa cells had been cotransfected with plasmids for constitutive RSV-promoter-driven Rep78 expression and for recombinant AAV vectors expressing a SV40-promoter-driven neomycin gene [42]. Furthermore, 1100 DNA sequences from a published analysis of *rep*-deficient AAV vector integration sites in diploid human cells [46] were reanalyzed. Since the PCR methods employed in our study and in the one by Drew et al. [42] cannot detect the matching left and right junction sites generated by one AAV integration event, only one chromosomal junction was analyzed per rescued provirus. The original DNA sequence files (DU711025.1 to DU709854.1) of Miller et al. [46] were downloaded from the Genome Survey Sequences (GSS) Database of NCBI (http://www.ncbi.nlm.nih.gov/sites/entrez?db=nucgss) and reanalyzed using the UCSC March 2006 human genome build. The analysis led to a total of 450 junction sequences that fulfilled all of the above inclusion criteria for bioinformatic comparisons. For the subsequent data analysis we implemented software in C++ using the software library SeqAn [74] and several Python scripts.

### Determination of distances of integration sites to putative Rep binding sites

For different Rep binding motifs, we computed the average distance of virus integration sites to the closest occurrences of Rep binding motifs within the genome. We supposed that the observed integration events were independent from each other and the sample size was high enough for assuming the distance to be normally distributed. To assess whether these distances differ significantly from expectation, several background models were generated:

1. For the background model “random”, we assumed that the probabilities for the observation of virus integrations were equally distributed among all conceivable positions in the genome. A program was implemented that computed the exact mean and standard deviation of this background distribution.
2. Since the integration site analysis required a suitable restriction enzyme site in the neighbourhood of the integrated virus three background-models for the restriction enzymes DraI, EcoRV or PvuII were generated. These models served as a corrective tool for an eventual bias of a non-uniform distribution of the respective restriction enzyme sites in the genome. For each AAV integration site observed, the distance to the closest restriction site was determined individually. Then, 1000 control sites per integration site were generated that displayed the same distance to randomly chosen restriction sites.

The generation of both, the data analysis and the background model was confined to those genomic contigs that contained at least one Rep binding motif, since otherwise the distance to the “closest Rep binding motif” would not be defined. A given set of AAV integration sites was considered to be significantly closer to Rep binding motifs than expected by chance, if the significance was calculated for *all* relevant background models. Data sets of AAV vectors were analyzed with the “random” background model. We applied a Z-test for determining statistical significances for the distances of integration sites to Rep binding sites. For comparing integration sites from AAV wildtype infection sites against those from rep-deficient AAV vector infection we applied the Student's t-test.

### Presence of genomic features

AAV integration sites were examined for the occurrence of various genomic features using tables available in the UCSC database. For the determination of significant divergences from expectations, we compared the actual integration sites with a set of 100,000 randomly chosen control sites in the human genome using a two-tailed binomial test.

### Analysis of chromatin state

Chromatin immunoprecipitation sequencing (ChIP-Seq) data were used to define the state of histone modifications in genomic regions of AAV integration. H3K27me3 domains determined by Cuddapah et al. were used as markers for closed chromatin (http://www.wip.ncbi.nlm.nih.gov/projects/geo/query/acc.cgi?acc=GSM325898) [75]. Regions enriched for H3K4 methylation (open chromatin) were determined as follows: The raw ChIP-Seq reads by Robertson et al. [76] (http://www.bcgsc.ca/data/histone-modification) were mapped to the human genome using Bowtie [77], and peaks were called using MACS [78]. H3K4me1/3 domains are then defined as 5 kb windows around the centers of the peaks.

## Supporting Information

Figure S1 Distribution of restriction sites in relation to Rep binding sites. (A) Cleavage sites of restriction enzymes used to digest genomic DNA of wildtype AAV-2-infected Hela cells and the numbers of occurrences per human genome are shown. (B) The mean distances of restriction enzyme cleavage sites to Rep binding sites were compared to those of random control sites to Rep Binding Sites. Calculations are displayed for consensus RBS that yielded significant proximity of integration sites to RBS as displayed in Figure 5. P-values were < 0.00002 for all motifs.
(0.13 MB TIF)

Figure S2 Bioinformatic analysis of AAV-2 wildtype integration sites. Distances of integration sites from Rep Binding Sites were calculated and the z-score was assessed in relation to the following control sites: (A) GAGC GAGC; (B) GAGT GAGC; (C) GAGY GAGC GAGC; (D) GAGC GAGC GAGC one mismatch allowed; (E) GAGC GAGC GAGC two mismatches allowed. In order to analyze only the Rep-binding sites outside of integration hotspot regions, sites within the hotspots of chr. 19 (AAVS1) and/or chr. 5 (AAVS2) were omitted in separate calculations.
(0.25 MB TIF)
