# Supplementary figures and images for: Integration Preferences of Wildtype AAV-2 for Consensus Rep-Binding Sites at Numerous Loci in the Human Genome
Source: PLoS Pathog. 2010 Jul 8;6(7):e1000985. doi: 10.1371/journal.ppat.1000985 (PMC2900306; doi:10.1371/journal.ppat.1000985)

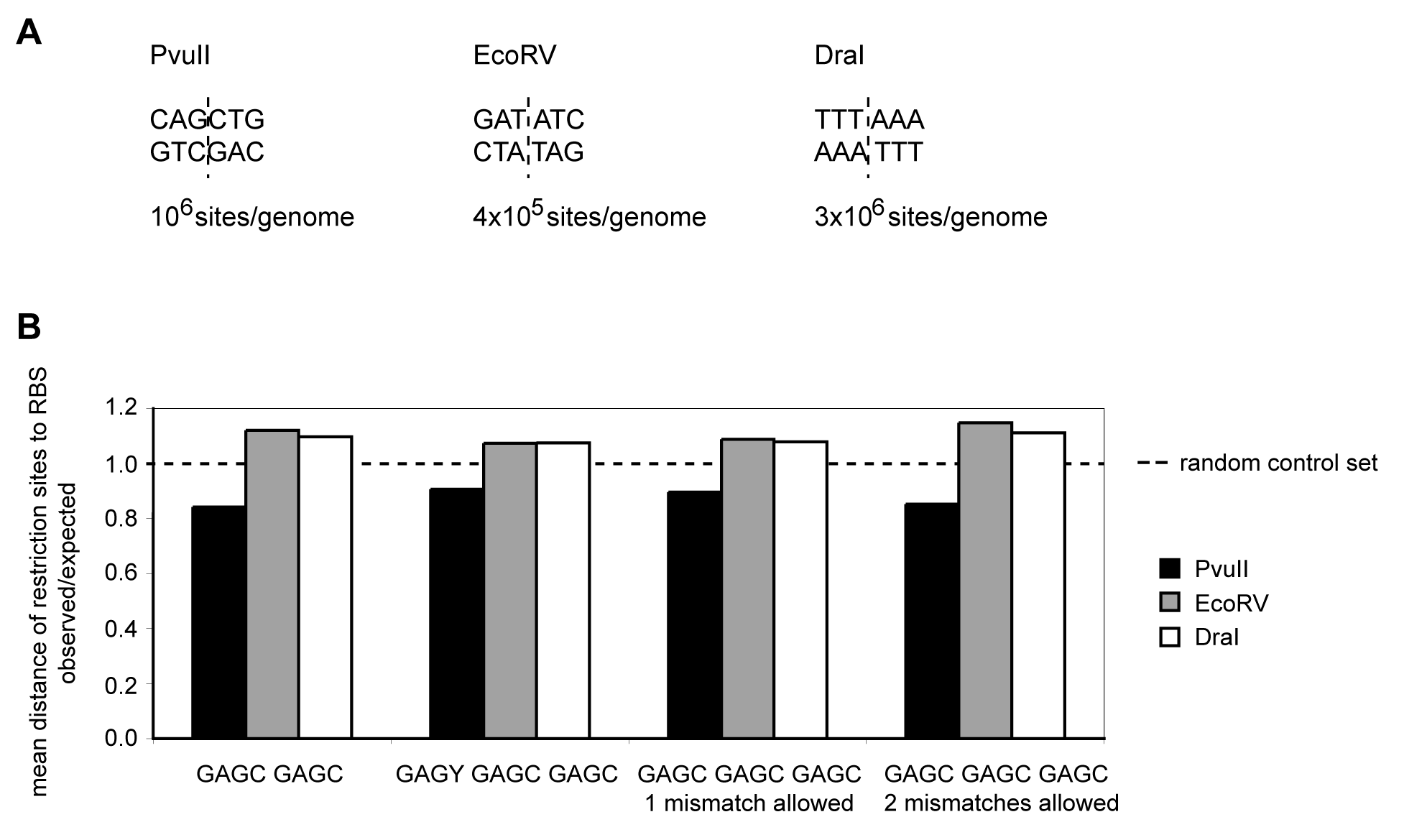

Supplement: Figure S1 — Distribution of restriction sites in relation to Rep binding sites. (A) Cleavage sites of restriction enzymes used to digest genomic DNA of wildtype AAV-2-infected Hela cells and the numbers of occurrences per human genome are shown. (B) The mean distances of restriction enzyme cleavage sites to Rep binding sites were compared to those of random control sites to Rep Binding Sites. Calculations are displayed for consensus RBS that yielded significant proximity of integration sites to RBS as displayed in Figure 5. P-values were < 0.00002 for all motifs. (0.13 MB TIF) [file ppat.1000985.s001.tif]

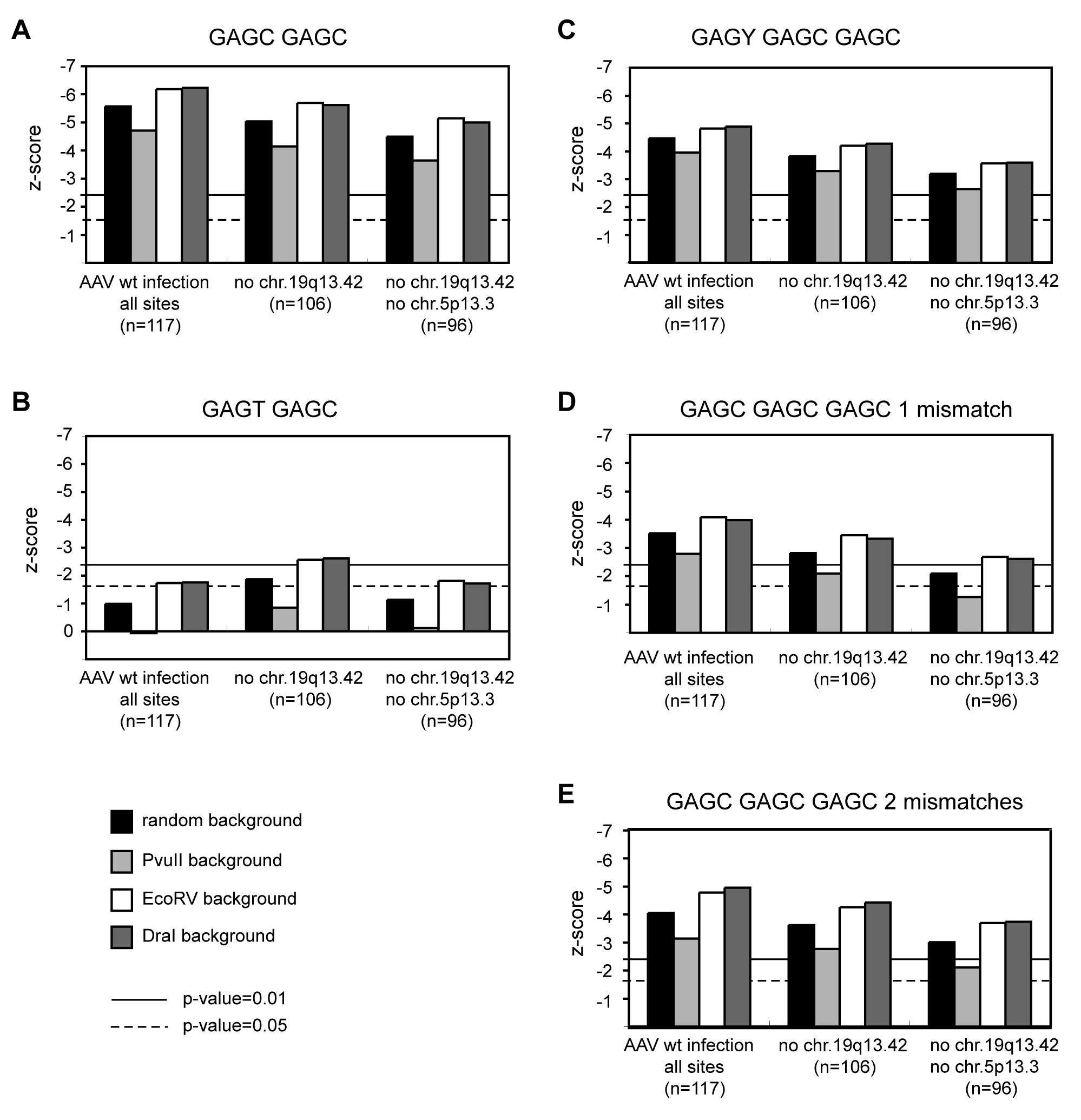

Supplement: Figure S2 — Bioinformatic analysis of AAV-2 wildtype integration sites. Distances of integration sites from Rep Binding Sites were calculated and the z-score was assessed in relation to the following control sites: (A) GAGC GAGC; (B) GAGT GAGC; (C) GAGY GAGC GAGC; (D) GAGC GAGC GAGC one mismatch allowed; (E) GAGC GAGC GAGC two mismatches allowed. In order to analyze only the Rep-binding sites outside of integration hotspot regions, sites within the hotspots of chr. 19 (AAVS1) and/or chr. 5 (AAVS2) were omitted in separate calculations. (0.25 MB TIF) [file ppat.1000985.s002.tif]
